# Revolutionizing cancer care with next-generation sequencing: an interview with Elaine Mardis

**DOI:** 10.1242/dmm.015396

**Published:** 2014-03

## Abstract

Elaine Mardis, co-director at the Washington University Genome Institute, has a long-standing interest in the development of sequencing technologies, which, in the 1990s, led her to play a pivotal role in the completion of the Human Genome project. Driven by the desire to apply her technological know-how to the improvement of human health, she then pioneered the sequencing and analysis of cancer genomes. These milestones have established Elaine as a leader in the cancer genomics field. In this interview, she recalls the events that shaped her career path, discusses the value of industry experience in a research setting, and provides her perspectives on challenges to clinical implementation of next-generation sequencing.

Elaine R. Mardis was born in 1962, in Nebraska, USA. Her undergraduate degree (Zoology) and PhD (Chemistry and Biochemistry) were obtained at the University of Oklahoma, where she had her first taste of molecular biology and became interested in DNA sequencing, under the mentorship of Bruce Roe. From 1989 to 1993 she was a senior research scientist at Bio-Rad Laboratories in Hercules, CA. In 1993, she joined the Genome Institute at Washington University in St Louis as, at that time, Director of Technology Development, and co-led a group that made a significant contribution to international efforts to sequence the human genome. Moving into the oncology field, she then helped to plan and coordinate a project that resulted in the first whole genome sequence of a tumor together with its matched normal genome and provided ground-breaking insights into the mutational basis of acute myeloid leukemia (AML). In recent years, she has continued to apply next-generation sequencing technologies to understand and treat cancer, and is heavily involved in The Cancer Genome Atlas (TCGA), a multi-institute initiative to catalog the genetic aberrations involved in cancers. The Genome Institute also studies human genetics as part of the 1000 Genomes Project, and assesses the druggability of the genome within the Pharmacogenomics Research Network. Still based in St Louis, Elaine is Professor of Genetics, with an adjunct appointment in the Department of Molecular Microbiology, and is co-director of the Institute. She serves on the scientific advisory boards of several biotechnological companies, including ZS Genetics, DNANexus, Qiagen/Ingenuity and GeneDX, and is a manuscript-handling editor for *Disease Models & Mechanisms*.

**Figure:**
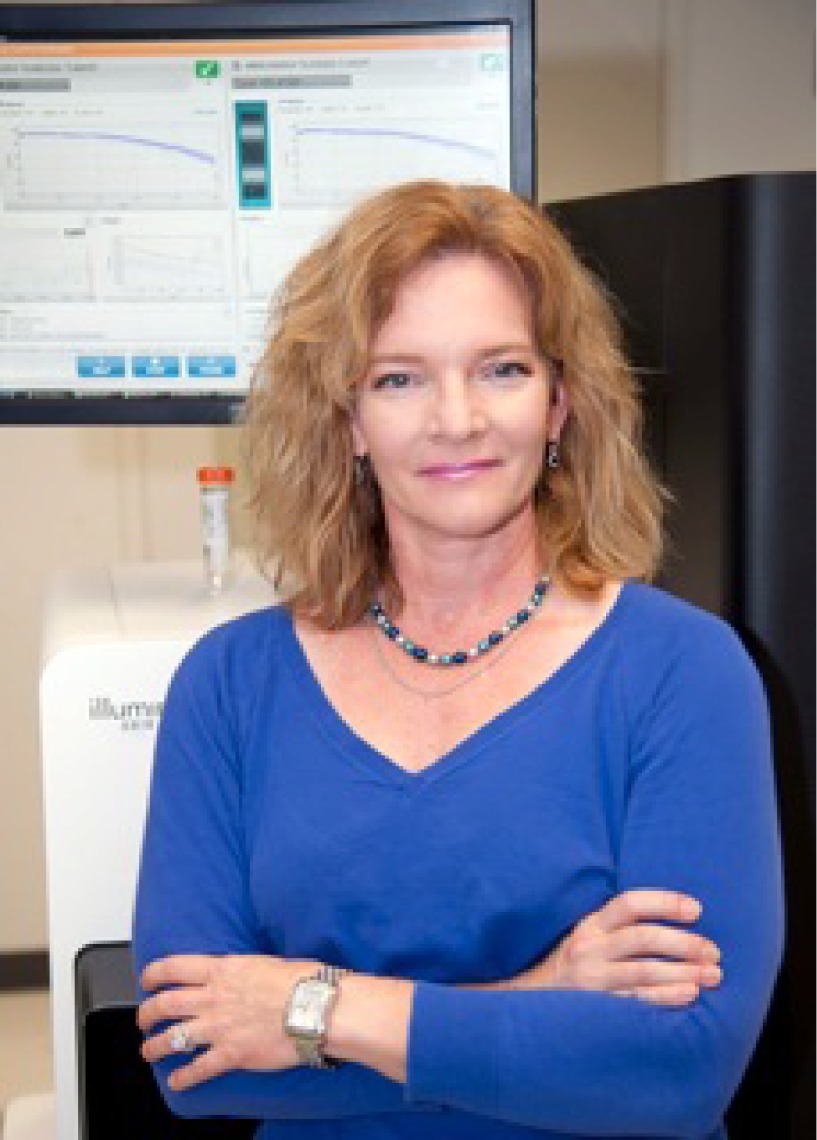


**Have you always been interested in science? Which mentors and experiences shaped your early research interests?**

My father was a chemistry professor. He kept old textbooks in the attic in our house and, as a child, I liked going up there to look at them. He had one that I particularly liked, on zoology, which had a picture of a duck-billed platypus – I remember that it fascinated me, and perhaps fuelled an early enthusiasm for science. In a cool turn of events, our lab ended up sequencing the platypus genome, many years afterwards!

My undergraduate degree was in Zoology and, for my research honors project, I focused on *Drosophila* genetics of mating behaviors. I worked with Dr Gerald Braver (now deceased) who was very far along in his career and close to retirement; he had trained with T. H. Morgan, perpetuating the long and fruitful scientific history of *Drosophila* genetics. Although it was a great experience to work with somebody of this calibre, I really didn’t have much of a feel for the project. It was too abstract for me to equate a gene I couldn’t see with complex mating behavior. I also had a bad habit of not watching closely when I anesthetized the flies – this was back in the days when we could still use ether – and often ended up killing them instead of only stunning them. This just added to my frustration. But, in my senior year at university I took a course in biochemistry that was taught by a professor named Bruce Roe. Bruce really opened my eyes to the world not just of biochemistry, but also, critically, of molecular biology. This just seemed so much more tangible and real to me, the way you can combine DNA and enzymes and things would happen and you could evaluate and actually *see* them. *Drosophila* geneticists would probably laugh and say that I was too stupid to get my head around the questions they address, and this might be true, but to me molecular biology just seemed so much more interesting.

“Bruce [Roe] really opened my eyes to the world not just of biochemistry, but also, critically, of molecular biology. This just seemed so much more tangible and real to me, the way you can combine DNA and enzymes and things would happen and you could evaluate and actually *see* them”

**Was it a natural next step to move towards molecular biology?**

I started talking to Bruce about my future and he convinced me to join his lab and get a PhD. This wasn’t my plan at the time, but I didn’t really have a firm alternative plan, and so it seemed a good option. I always tell people that I’ve just two degrees of separation from Fred Sanger, because Bruce learned sequencing while on sabbatical in the Sanger lab, and of course I then learned DNA sequencing from Bruce. Rick [Richard] Wilson, who I’ve worked with for over 20 years, was also a trainee in Bruce’s lab, so we come from the same background and have enjoyed a close scientific partnership since meeting in Bruce’s lab.

Bruce was a key mentor, and I talk to him to this day. He is semiretired but still very much in the know, and any time we have a big headline or paper, I always get a nice email from him.

**What alternative path might you have taken, if you hadn’t decided to do a PhD?**

I always planned to do something related to science but at the time I wasn’t sure I had the kind of energy needed to make it through another 5 years of college. Also, I was a bit naive as to what graduate school and getting a PhD entailed. When I first started talking with Bruce about postgraduate studies, what I was really thinking about doing – bear in mind that this was back in the mid-1980s and the human genome was of interest but hadn’t really leapt to the forefront – was getting a Master’s degree in genetic counselling. In my mind, 2 years was better than 5 or 6 years of additional school, and I viewed genetic counselling as a great practical application of genetics in the clinical setting. Bruce was supportive of this, and said that he would write me a letter of recommendation, but at the same time he said that I might get more out of doing a PhD. It ended up being the better option for me, as he predicted.

**How did your experience in industry impact on your research career? Would you say that some of the industry experience that you brought to Washington University contributed to the success that the Institute had in sequencing the human genome?**

I finished my PhD in 1989, when the notion of sequencing the human genome was just coming into play. Although I’d spent most of my PhD very focused on DNA sequencing and the development and automation of methods (all the things that I later ended up doing at Washington University), it wasn’t really the time to go forward with this. There was a lot going on in the realm of physical mapping: trying to take pieces of chromosomes and make sense out of them. Despite understanding that physical mapping was a necessary intermediate to properly organizing the sequencing of the genome, I had zero interest in it, so opted to do more technology development work. Industry was the logical step, and I took an opportunity at Bio-Rad, which back then was doing a lot in the automation of DNA sequencing, and was actively coming on board with DNA sequencing reagents. I viewed it as a useful training opportunity, which it was, but it also turned out to be a very valuable experience on two very practical levels. First, it got me into a setting where there were tiers of management, and for the first time I was really able to experience being a manager as well as a scientist. If you end up being a good manager of your lab once you’re a PI that’s great, but many scientists are poor managers. I had the opportunity to evaluate what real management was like and how to deal quickly with problems in terms of personnel, such as disputes and dissatisfaction, to avoid letting these problems fester. This served me well over time, because at the peak of the human genome project, our group was made up of almost 300 people. At this scale, a tier of management is essential.

The second skill I learned, which was again important later when we grew the scale of operations, was how to orchestrate proper manufacturing procedures, including QA and QC [quality assurance and quality control]. Again in a historical context, when we first started scaling up our efforts to sequence the *C. elegans* genome and then the human genome, we didn’t have manufacturers like, for example, Illumina who made bulk reagents that you could just put in the machine, add your tubes, punch the right buttons and walk away. When I originally joined the lab in 1993, every day the sequencing technicians manually put together the sequencing reagents in a cocktail. Of course, people can have bad days, and sometimes things were left out; so, you would have C, G and T but no A and the reaction failed. As we scaled up, we quickly realized we needed to turn ours into more of a manufacturing-like setting. Taking my experience from industry, I set up a core group that was responsible for manufacturing the sequencing reagents, buffers and other necessary solutions, such as for DNA isolation. In addition, I implemented QC protocols so that we knew there was quality and consistency across batches. Without the industry experience, I wouldn’t have had a clue where to start. Granted, I could have figured it out, but the prior knowledge meant we were able to hit the ground running, and we quickly attained a level of reproducibility and certainty.

I would love to take credit for everything wonderful that happened at the Genome Center in the 1990s. I contributed, but it was a huge team effort and a lot of organizational factors came into play. The nucleus of people that were there at the time was crucial, with Bob Waterston leading the group overall, Rick and I, and John McPherson who was absolutely integral to getting the physical map of BAC clones figured out for the Human Genome Project. Also, LaDeana Hillier ran our bioinformatics group and played an incredibly crucial role in our successes in mapping and sequencing, and I enjoy working with her to this day. I think it was a collective effort where we all made strong contributions that overall led us to be very successful.

**What is the next stage in the human genome sequencing project?**

To this day, Washington University continues to contribute to the completion of the human genome, by filling in the gaps and monitoring and including all of the new content. We work very closely with the NCBI [National Center for Biotechnology Information] to continually refine the reference genome. Frankly, I think these efforts go underappreciated because success is hugely dependent upon the reference sequence being of high quality and an increasingly accurate representation of the human genome. The more human genomes we sequence, the more we realize that the reference is valuable but still pretty inadequate in some ways. We recently completed a proposal to NHGRI [National Human Genome Research Institute] to again put much more effort into the project: to sequence and finish additional human reference genomes so that we can have a much fuller picture of content and diversity for multiple human genomes.

**When did you first become interested in applied science, and how did you pick up the language of oncology so quickly?**

We’ve known since the 1970s when Janet Rowley and others starting looking under the microscope at chromosomes that there is clearly something genomically different about cancer cells. In a way, we now have a very finely tuned ‘microscope’ that looks at DNA at the single-nucleotide resolution, but ultimately tells us the exact same thing. People lose sight of the fact that the major purpose behind the Human Genome Project was to understand the cause of human disease. Once we had a finished blueprint, this was the natural direction to move in.

My first foray into cancer came in 2003–2004, in partnership with Harold Varmus and his young trainee William Pao, both then at Memorial Sloan Kettering. At that point in time, a small proportion of lung cancer patients were being given new drugs called tyrosine kinase inhibitors, and about 10% of these patients were experiencing a tremendous benefit. That a drug could clear lung lesions in metastatic patients within a few short weeks was just miraculous. However, there was the question of the other 90% who weren’t responding. We took a handful of patients from Harold and William’s studies of tyrosine kinases and lung cancer and said: ‘Right, we have a limited ability to look at the genome – let’s find out what’s different about tyrosine kinase genes in these patients who are responding versus those who don’t’. We knew that there are key tyrosine kinases that act as ‘drivers’ in cancer, and we inferred that these are probably the ones being targeted by tyrosine kinase inhibitor drugs. Sequencing revealed that patients who showed a dramatic response to these drugs had a key series of mutations throughout the tyrosine kinase region of epidermal growth factor receptor. We reported this coincident with two other groups, and so all the papers got published together. It was really our first experience of doing something great and tangible with the human reference genome we had helped to sequence.

Another contributor to my training in cancer care was afforded by the role I played as Basic and Translational Sciences Director for the American College of Surgeons Oncology Group, one of the NCI [National Cancer Institute]-funded cooperative groups. This exposure to the organization, conductance and analysis of clinical trials was incredibly important in helping me to understand basic concepts of oncology, clinical care and standard practice. I also learned how drugs are approved for use in patients, and about the basics of clinical trial design. I was further rewarded in this experience by being able to include samples from ACOSOG clinical trials in some of the genomics studies we have done at my institute.

I recall that when I started working within ACOSOG, I felt like I knew nothing about cancer, clinical trials, clinical care paradigms and how genomics would ‘fit’ into this arena. Nowadays, I’m feeling much more comfortable in the cancer genomics space, mainly because I’ve had the chance to be involved with a lot of the key people who are leading this area. It’s one of the real advantages of being at Washington University – I have great clinical collaborators who really understand the power of genomics.

**What’s the history behind the milestone 2008 paper in which you reported whole genome sequencing of a tumor genome?**

Between 2005 and 2007, before next-generation sequencing [NGS] came along, we were working on AML in collaboration with Timothy Ley. Sequencing was still really expensive, and so we were working on only the candidate genes using PCR. We had some insights, but never an ‘aha’ moment in terms of finding out what drives the pathogenesis of AML, which was the fundamental question. Along came the 454 sequencer [now produced by Roche] followed by the Solexa sequencer. The potential of the Solexa sequencer, realized during our early access testing, inspired a discussion one day over lunch, where Tim, Rick and I cooked up the idea of sequencing the whole genome of a cancer patient. We thought we could map the tumor and matched normal genomes back to the reference to start figuring out what was different. One of our statisticians worked out how many runs of the sequencer would be needed to get the whole genome, and came up with a notion of 30-fold coverage. These numbers allowed us to calculate what it would cost to do whole genome sequencing on a small number of patient samples. It was crazily expensive, but we hedged our bets and proposed it to NCI in the renewal of a program project grant. Of course it was considered ridiculously ‘too early’ in terms of technology and informatics; it got shot down in study section review, and they were incredulous that we would even suggest it.

We then went to a local philanthropist, Alvin J. Siteman, in St Louis; we got a meeting with him through someone who shall remain nameless that was very high up in the Medical School organization. We sat down with him, and basically in a 1-hour meeting tried to convince him that this was the next great step in cancer research. He said he was going to think about it and get back to us by the next day. When that day came, we had a million dollars’ worth of stock shares that he instructed us to cash in to give us our million dollars – this was the golden number we had calculated to do the whole genome of one AML patient, tumor and normal. In reality, it probably cost a little bit more, because we had no idea how to do the bioinformatics. We worked and worked at the analysis; nowadays, we don’t analyze data for somatic mutations in the same manner we did that first time, but we were learning as we went along. We identified ten coding mutations, which we thought seemed like a small number. However, fast-forwarding to the recent TCGA paper that looked at 200 AMLs across subtypes, that number is largely unchanged (the average number of mutations across all subtypes was 12). So we were pretty close to right, which is gratifying in retrospect.

So, this is how it all started, with the confluence of a great idea, good clinical samples for which we had full information on how the patient had been treated, and then the 800 lb gorilla in the room – the Solexa instrument that rapidly accelerated the rate at which we could sequence a genome. It’s all about timing, and everything fell into place together at the right time.

**Sequencing has obviously become cheaper and more efficient since then. What are the key clinical applications of NGS?**

Cancer is the easiest and most obvious one, with the caveat that it is neither easy nor obvious at times. For cancer diagnoses, our group plans to apply what I call the ‘Maserati’ approach – a three-pronged approach in which the whole genome of tumor and normal is combined with information at the exome (tumor and normal) and transcriptome (tumor only) levels, to give us the most comprehensive, in-depth view of a patient’s somatic landscape. You would be surprised at how often there’s no DNA-based evidence for overexpression of a druggable gene, so it’s important to integrate genomic and expression data to get the fullest picture of what could work for a patient. There are other groups using NGS in the cancer clinical care realm, and I feel strongly that this year’s [2014] activities either will help us make the case for clinical efficacy by this approach, or show that we still don’t know enough to do it well enough to benefit patients.

The other clear benefit of NGS in the clinic, evidenced by some nice reports in the literature, is to diagnose children with rare diseases. In some cases, it’s not only the genetic lesion that is identified, but also a drug that addresses that lesion, which is incredible. For parents of a child that is having difficulty getting a diagnosis, the notion that you can look at the genome, potentially identify the causative mutation or mutations and then examine those loci in the parents is also helpful for them to understand whether this is a one-off or whether it has a high probability of happening again with the next child. On the other hand, for common, complex diseases, there frankly hasn’t been a lot to crow about. We’ve confirmed things that we already knew, which is valuable, but I would argue that we need to take a pause on teasing out the nuances of the genome to start really understanding more of its functionality in disease, including the non-coding regions of the genome.

There have been some gains due to NGS in family studies of neural developmental disorders; autism is probably one of the best examples. However, a lot of complex neurological diseases appear not to be caused by point mutations, but by large structural changes such as duplications or deletions. So, I think we will need whole genome sequencing to become much less expensive before we can begin to have a complete picture of all the contributing alterations. Although NGS is still not the perfect technology to look at these larger changes, I think we have made great strides in understanding very complex regions of our genome.

**Are animal models an important aspect of this?**

It sounds gratuitous but I think animal models remain incredibly important, particularly in translational research. It just can’t be overstated how many things you can do in an animal-model setting to replicate human disease, and also to manipulate it. Most would agree that we have to continue to fund and perpetuate model organism research. Despite this, there are some experiments, and I use that term loosely in this context, that you want to do with human samples. For example, some of the most valuable metastatic specimens that we can obtain in a solid-tumor setting are going to be those from patients who consent prior to death to an autopsy, followed by banking of that material. I think the challenge lies in banking material from patients as close to death as possible, just thereafter of course. But, in the US at least, there are not a lot of banking autopsies performed and it is really too bad: I would like to see more efforts to change this.

“I think animal models remain incredibly important, particularly in translational research. It just can’t be overstated how many things you can do in an animal-model setting to replicate human disease, and also to manipulate it”

**What would you say are the main challenges to the implementation of NGS in routine clinical practice?**

I think the main challenge lies in the analysis of sequencing data. We miss a lot and we misinterpret a lot so we get a high level of false positives and a high level of false negatives. It’s a tremendous ongoing effort to come up with hardened bioinformatic pipelines that evaluate NGS data in the most sensitive way possible, with the lowest percentage of false positives and false negatives. Again, the value of including RNA sequencing for correlative evidence is very powerful in improving precision. Most DNA-based diagnostics nowadays involve Sanger sequencing, which clinicians are comfortable with, and we need to really establish that what we are getting from next-generation analysis is in many ways better, but at least equivalent. Although people commonly refer to Sanger sequencing as the gold standard, it falls apart in some ways that NGS can compensate for. For example, NGS provides greater sensitivity to detect minor variants at high depth of sequencing coverage, copy number estimation and a feel for heterogeneity of a given biopsy. That said, NGS is not the perfect technology because the reads are still too short and the analysis is still too complicated around some of the regions of the genome that you also can’t analyze by Sanger. Because of these complications both at the level of Sanger and at the level of NGS, I feel there is still a lot to be done to get this equivalency established.

Another challenge is working to the right scale. NGS by its nature generates large data sets, and we struggle to bring it down to small, discrete regions of the genome. That’s a technological challenge that people are trying to address. There is a dichotomy because you want to ask unbiased questions but the data sets are too large and informatics is too complicated to be clinically relevant, so instead you settle for smaller questions but you are then challenged from a breadth standpoint. This is a problem when looking at cancer, because the disease itself takes on so many forms and flavors of somatic alterations for each patient that you can’t really get a comprehensive picture by taking a tiny snapshot of the genomic landscape. So, we favor a broad-based approach. The clinical implementation looks very real and we have tangible evidence that it can pan out, but the technological and bioinformatics challenges are continually locking horns, determining what exactly we can do and how.

“The clinical implementation looks very real and we have tangible evidence that it can pan out, but the technological and bioinformatics challenges are continually locking horns, determining what exactly we can do and how”

**What advice do you give to young scientists hoping to follow in your footsteps in terms of technological development and its applications in translational research?**

I often advise students to include two areas – bioinformatics and statistics – in their training. I don’t have any aptitude for either of these areas, but I’ve come to realize that they are incredibly important. If you are trained in high-energy physics you learn not only how to set up experiments but also how to interpret the data, and I feel we can learn from this in biology. I emphasize to young researchers that NGS is exciting, but if you can’t analyze the data you can’t do anything with it – you can’t learn from it. I am involved in teaching a course at Cold Spring Harbor that is focused on NGS and its applications. In this course, we absolutely insist that even those who come in with a biology background need to understand data analysis by the time they leave. We spend the first 4 or 5 days on the how-to of making libraries, generating sequencing data, and then we spend the rest of the 2½ weeks just taking a deep dive on analysis of different data types – RNA, hybrid capture sequencing, methylation analysis – and we show the students how the analysis gets done. As well as the data analysis, you need to have at least a rudimentary understanding of statistics to really make sense of data and understand experimental design as well as possible.

“In the past, you could end up with an illustrious career working independently, but I think we’re now in a very different era. To have maximum impact, we have to be in a collaborative setting and we have to be collaborative”

The other thing I tell students is to learn how to be a good collaborator and respect the contributions that other people bring. It’s going to take large groups made up of people with different areas of expertise to come up with answers that can be applied to human health. If you can’t get along and respect what other people bring to the table, you’re not going to be deemed a good collaborator and you are not going to be invited to participate. It sounds obvious, but I think sometimes science, particularly biology, can have some singularity to it. In the past, you could end up with an illustrious career working independently, but I think we’re now in a very different era. To have maximum impact, we have to be in a collaborative setting and we have to be collaborative.

**How do you relax outside of the lab?**

My best relaxation nowadays comes from cooking, which I really enjoy. I also practise martial arts a little, but not as much as I probably should! On rare occasions, I get out my golf clubs and go for a round of golf. I also go to a lot of meetings, and this is actually relaxing for me – I’m a scientist, I love science, and I love to talk about it pretty much all the time. I really enjoy the shared enthusiasm and purpose, and the excitement of taking a technology advantage and starting to apply it to patient care. I probably would have pursued a medical degree if it weren’t for the fact that I can’t stand the sight of blood. This is as close as I’m going to get to that, and it’s incredibly exciting.

